# *Phlogacanthus pulcherrimus* Leaf Extract as a Functional Feed Additive: Influences on Growth Indices, Bacterial Challenge Survival, and Expression of Immune-, Growth-, and Antioxidant-Related Genes in *Labeo chrysophekadion* (Bleeker, 1849)

**DOI:** 10.3390/life15081220

**Published:** 2025-08-01

**Authors:** Sontaya Sookying, Panitnart Auputinan, Dutrudi Panprommin, Paiboon Panase

**Affiliations:** 1 Division of Pharmacy and Technology, Department of Pharmaceutical Care, School of Pharmaceutical Sciences, University of Phayao, Mueang, Phayao 56000, Thailand; sontaya.so@up.ac.th; 2 Division of Biotechnology, School of Agriculture and Natural Resources, University of Phayao, Mueang, Phayao 56000, Thailand; panitnart.au@up.ac.th; 3 Division of Fisheries, School of Agriculture and Natural Resources, University of Phayao, Mueang, Phayao 56000, Thailand; dutrudeep@yahoo.com; 4 Unit of Excellence “Physiology and Sustainable Production of Terrestrial and Aquatic Animals”, Division of Fisheries, School of Agriculture and Natural Resources, University of Phayao, Mueang, Phayao 56000, Thailand

**Keywords:** gene expression, growth performance, immunity, *Labeo chrysophekadion*, *Phlogacanthus pulcherrimus* extract, phytochemical composition

## Abstract

This research examined the impact of dietary supplementation with *Phlogacanthus pulcherrimus* extract (PPE) on the growth, disease resistance, and expression of immune-, growth-, and antioxidant-related genes in *Labeo chrysophekadion*. Over 150 days, 90 fish from each group were fed diets with 0 (control), 0.25, 0.50, or 0.75 g/kg of PPE. Phytochemical analysis revealed phenolics (96.00 mg GAE/g), flavonoids (17.55 mg QE/g), anthraquinones, and triterpenoids, along with moderate antioxidant activity (IC_50_ = 1314.08 μg/mL). One-way ANOVA of growth indices, including weight gain, specific growth rate, feed conversion ratio, and survival rate, revealed no significant differences (*p* > 0.05); however, PPE supplementation significantly enhanced immune and antioxidant gene expression. *IL-1β* was significantly (*p* < 0.05) upregulated at all doses, with the highest expression observed at 0.50 g/kg, showing a fivefold increase compared to the control. In addition, the highest relative expressions of *IGF-1* and *CAT* were found at 0.75 g/kg, with 4.5-fold and 3.5-fold increases compared to the control, respectively. PPE at 0.75 g/kg decreased the cumulative mortality rate (CMR) by 20% compared to the control group, which had a CMR of 50% following exposure to *Aeromonas hydrophila*. PPE acted as an effective immunostimulant and antioxidant, supporting reduced antibiotic reliance in aquaculture.

## 1. Introduction

Black sharkminnow (*Labeo chrysophekadion* (Bleeker, 1849)) (family Cyprinidae) is a freshwater fish commonly found in both stagnant and flowing waters across Southeast Asia. Its natural distribution includes the Mekong and Chao Phraya basins, the Malay Peninsula, Sumatra, Java, and Borneo [1]. This species is highly valued for consumption due to its large body size, substantial flesh content, and palatable taste [2]. It is recognized as an economically important aquatic species in several Asian countries, including Thailand, Laos, Vietnam, and Cambodia. Nonetheless, the natural populations of *L. chrysophekadion* are currently declining, primarily because of environmental degradation that adversely affects their reproductive capabilities and habitat quality. Although breeding technologies under controlled conditions for this species have been successfully developed [3], the market demand for black sharkminnow remains high. Apart from being consumed as food, it is also popular as an ornamental fish [2,4], contributing to its relatively high market price. However, the only available export data for this species from Thailand pertains to its status as an ornamental fish, with statistics recorded from 2018 to 2020. During this period, approximately 60,000 individuals were exported, with an estimated value of around THB 335,557 (equivalent to approximately USD 10,000) [5].

*Phlogacanthus pulcherrimus* T. Anderson (family Acanthaceae), commonly known as “Dee Pla Kang” in Thai, is a widely distributed medicinal and culinary plant, particularly in northern and northeastern Thailand. In Thai traditional medicine, it has been used as a general tonic, diuretic [6], and appetite stimulant [6,7]. *P. pulcherrimus* is commonly found in the wild and is also cultivated in home gardens in some regions [7]. Remarkably, reports show a 100% survival rate when wild plants are transplanted for cultivation in the greenhouse condition [8], suggesting that *P. pulcherrimus* is a native, non-toxic, easily cultivated food plant with no known adverse effects on humans or the environment.

Beyond its traditional uses, recent studies have revealed additional pharmacological activities of *P. pulcherrimus*. Boontha et al. [9] reported that ethanol extracts of the leaves exhibit anticancer activity against MCF-7 breast cancer cells through multiple mechanisms. Further research by Kheawchaum et al. [10] identified diterpenoid lactone glycosides and phenolic glycosides from *P. pulcherrimus* with cytotoxic activity against various cancer cell lines, including T-lymphoblast (MOLT-3), hepatocellular carcinoma (HepG2), and cervical cancer (HeLa). Notably, only one out of twenty compounds showed low-level toxicity against normal MRC-5 cells [10]. The safety of *P. pulcherrimus* extracts in normal Vero cells was confirmed by Athipornchai et al. [11].

The plant also exhibits strong inhibitory activity against alpha-glucosidase, a key enzyme in carbohydrate digestion that influences blood glucose levels and reflects physiological stress. The leaf extracts of *P. pulcherrimus* significantly outperformed the standard drug acarbose in α-glucosidase inhibition assays, as confirmed by several studies [11,12,13]. In addition to its pharmacological properties, the nutritional composition of *P. pulcherrimus* has also been analyzed. Chaichana et al. reported that the plant is rich in minerals—particularly calcium, iron, magnesium, and zinc—and contains protein, fat, carbohydrates, and dietary fiber at levels of 22.5%, 4.2%, 41.1%, and 16.3%, respectively [14]. The high magnesium and zinc content may contribute to its antioxidant and biological activities, complementing its phytochemical profile, including flavonoids and phenolic compounds.

The application of herbal extracts as dietary supplements in aquaculture has garnered increasing scientific attention due to their potential benefits, including antimicrobial and antifungal activities, immune system enhancement, stress alleviation, improved growth performance, decreased dependence on chemical treatments, and alignment with consumer preferences and ongoing research innovations [15,16,17]. Given the high market demand for *L. chrysophekadion* both as a food source and as an ornamental fish amidst the declining wild populations and reproductive challenges, the pharmacological potential, non-toxicity, and ease of cultivation of *P. pulcherrimus* is of scientific interest.

Based on the literature review, there are no prior reports on the use of this plant extract in *L. chrysophekadion*. Consequently, this study aims to investigate the effects of ethanol leaf extract of *P. pulcherrimus* on growth performance and the expression of key genes, including interleukin-1β (*IL-1β*), insulin-like growth factor 1 (*IGF-1*), and catalase (*CAT*) of black sharkminnow. These genes were selected due to their fundamental roles in immune response, growth regulation, and oxidative stress defense, respectively.

## 2. Materials and Methods

### 2.1. Ethical Considerations and Animal Use Authorization

All experimental procedures were approved by the Committee of Institutional Animal Care, University of Phayao, Thailand (approval ID: 1-010-67; date of approval: 1 August 2024), and were performed in compliance with the institutional guidelines. Authorization to use laboratory animals was granted to the researcher, Paiboon Panase, under license No. Ul-01217-2558 by the National Research Council of Thailand.

### 2.2. Plant Materials and Chemicals

Fresh leaves of *P. pulcherrimus* were collected from natural areas in Phayao Province and purchased from a reliable local supplier. A voucher specimen was prepared by pressing and drying the plant material, which was subsequently identified by a botanical taxonomist. The specimen was deposited at the Queen Sirikit Botanic Garden (QBG) Herbarium, with voucher number 149,658.

All chemicals used in the experiments were of analytical grade. Ethanol (99.9%) and methanol (99.9%) were obtained from RCI Labscan, Dublin, Ireland. Aluminum chloride hexahydrate (95%) and sodium carbonate anhydrous (99.5%) were sourced from KemAus, Sydney, Australia. Sodium hydroxide (99%) was acquired from RCI Labscan, Ireland. Gallic acid (98%) was supplied by AK Scientific, Union City, CA, USA. Quercetin dihydrate (98%) and L-ascorbic acid (99.7%) originated from Sisco Research Laboratories, Mumbai, India. Folin–Ciocalteu’s phenol reagent was purchased from Loba Chemie, Mumbai, India, and 2,2-diphenyl-1-picrylhydrazyl (DPPH) was supplied by Sigma-Aldrich, Waltham, MA, USA. Reagents for phytochemical screening tests were reagents of analytical grade and were supported by the School of Pharmaceutical Sciences, University of Phayao.

### 2.3. Preparation of P. pulcherrimus Extract

Fresh leaves of *P. pulcherrimus* at the intermediate growth stage (neither too young nor too mature), exhibiting intact morphology and free from diseases and insect damage [13], were thoroughly washed and dried in a hot-air oven at a temperature not exceeding 45 °C. The dried leaves were ground using a grinder and sieved through a 40-mesh sieve to obtain a powder with a particle size of 400 μm. A total of 500 g of dried leaf powder was weighed and macerated with 95% ethanol at a ratio of 1:4 (*w*/*v*). The mixture was continuously agitated using a shaker at 100 rpm for 72 h. Upon completion of the extraction period, the mixture was filtered through Whatman no. 4 filter paper with a pore size of 20–25 μm, in combination with a Buchner funnel and suction flask, and concentrated to dryness using a rotary evaporator. The resulting PPE was stored in a tightly sealed, light-protected container at a temperature not exceeding 8 °C until further use [18].

### 2.4. Phytochemical Screening of PPE

In this investigation, the phytochemical profile of PPE was examined using standardized protocols, as outlined by Pant et al., Hartanti and Cahyani, and Shaikh and Patil [19,20,21]. The presence of alkaloids was assessed using Dragendorff’s and Valser’s reagents. To evaluate steroidal and terpenoid components, the Liebermann–Burchard test was employed. Molisch and Keller–Killiani assays were used to detect carbohydrates and deoxy sugars, respectively, while α,β-unsaturated five-membered lactone rings were identified via the Kedde test. Cyanogenic glycosides were screened using the sodium picrate technique, and the frothing method was utilized to detect saponins. Ferric chloride reagent was applied to identify phenolic compounds, whereas flavonoids were confirmed using Shinoda’s test. Tannins and condensed tannins were indicated by reactions with gelatin solution and bromine water, respectively. The sodium hydroxide paper method was used for detecting coumarins, and anthraquinones were determined through a modified Borntrager’s procedure. Lastly, the presence of anthocyanins was established via acid–base indicator testing.

### 2.5. Determination of Total Phenolic Content in PPE

The total phenolic content of the extract was quantified using the Folin–Ciocalteu colorimetric method, as adapted with minor modifications from the procedure described by Ref. [22]. Gallic acid served as the reference standard, with a calibration curve prepared over the concentration range of 3.125–200 μg/mL. Both gallic acid and PPE stock and working solutions were prepared using 80% methanol as the solvent. For the assay, 20 μL of either the standard or the extract was dispensed into a 96-well microplate. Subsequently, 50 μL of 10% Folin–Ciocalteu reagent (diluted in water) was added. After gentle mixing, the mixture was incubated in the dark for 5 min. Then, 80 μL of 7.5% sodium carbonate solution was introduced into each well. The reaction was allowed to proceed in the dark for 30 min before measuring absorbance at 760 nm. The calibration curve generated from gallic acid standards followed the linear equation y = 0.0044x + 0.0041, with a coefficient of determination (R^2^) of 0.9958. The phenolic content was expressed as milligrams of gallic acid equivalents per gram of extract (mg GAE/g extract). All measurements were performed in triplicate to ensure accuracy and reproducibility.

### 2.6. Determination of Total Flavonoid Content in PPE

The total flavonoid content was assessed using a modified aluminum chloride colorimetric assay based on the method described by Ref. [22]. Quercetin was employed as the standard compound, and both quercetin and PPE solutions were prepared in 80% methanol. A calibration curve was established using quercetin concentrations ranging from 3.125 to 200 μg/mL. For the assay, 20 μL of each standard or test sample was dispensed into a 96-well microplate, followed by the addition of 50 μL of 2% aqueous aluminum chloride. After a 5 min incubation in the dark, 50 μL of 50 mM sodium hydroxide solution was added. The mixture was then further incubated in the dark for 30 min, and the absorbance was recorded at 415 nm. Flavonoid content was quantified based on the quercetin calibration curve (y = 0.0037x + 0.0011, R^2^ = 0.9999) and expressed as milligrams of quercetin equivalents per gram of extract (mg QE/g extract). All analyses were performed in triplicate to ensure data reliability.

### 2.7. Determination of Antioxidant Capacity of PPE

The antioxidant capacity of PPE was evaluated using the DPPH radical scavenging assay, with slight modifications from the method of Ref. [23]. Stock solutions of DPPH (0.1 mM) and ascorbic acid (2000 μg/mL) were prepared in methanol, while working solutions of ascorbic acid and PPE were diluted with 80% methanol. In a 96-well plate, 100 μL of sample or standard was mixed with 100 μL of DPPH solution. After shaking and incubating in the dark for 30 min, absorbance was measured at 517 nm. Radical scavenging activity was calculated as % inhibition = [(A_blank_ − A_sample_)/A_blank_] × 100 and plotted against concentration to determine the IC_50_ value, where A_blank_ and A_sample_ are the absorption of the blank and sample, respectively. All measurements were performed in triplicate.

### 2.8. Experimental Fish and Acclimatization Conditions

The protocols for this research involved procuring juvenile *L. chrysophekadion* from a local aquaculture facility in Phayao province, Thailand. All the fish were transferred to a net cage (5 m × 3 m × 2 m) situated in the earthen ponds and acclimatized for four weeks under a natural photo period. Throughout the acclimatization phase, the monitored water quality metrics were as follows: The temperature was maintained at 28.6 ± 3.18 °C, the dissolved oxygen (DO) content was 6.50 ± 1.8 mg/L, and the pH was 7.8 ± 1.52 (multi-probes, HORIBA, U50 series, Kyoto, Kapan). During the acclimatization phase, the fish were fed twice daily (8 a.m. and 5 p.m.) with commercial fish feed at a rate of 4% of their body weight per day (40% crude protein); at this stage, their diet did not include any PPE.

### 2.9. Preparation of a PPE-Supplemented Diet

During the investigation, 2 mm-diameter commercial fish feed pellets (Hi-grade 9961, CPF Co., Ltd., Bangkok, Thailand) were utilized, including 40% crude protein, 4% fat, 12% moisture, and 4% fiber. Commercial fish feed comprises fish meal, soybean meal, maize, broken rice, vitamins, and minerals. The PPE was dissolved in 100 mL of distilled water. The feed was categorized into four groups: a control group (0.00) that did not receive PPE (T1) and three experimental groups treated with varying concentrations of 0.25 (T2), 0.50 (T3), and 0.75 (T4) g/kg, respectively. The substance was meticulously blended using a pan coating machine with an air blower (CM/Thai, CMCA-10 model, Pharmaceutical and Medical Supply Co., Ltd., Samut Sakhon, Thailand). Subsequently, the pellets from all four groups were covered with a 4% agar solution at a rate of 10 mL/kg and then air-dried once more [24]. Subsequently, the combined fish meal quantities were placed individually in sterile containers at ambient temperature. This approach was executed weekly, after which the blended diet was promptly ingested by the fish.

### 2.10. Experimental Design

Following acclimatization, healthy fish with an average body weight of 0.89 ± 0.52 g were allocated to 12 net cages (1 m × 2 m × 0.8 m; mesh size, 2.5 mm^2^) arranged in four triplicate groups. The stocking density was 30 fish per net cage (19 fish per m^2^), and all groups were assigned to the same earthen pond. All groups administered a diet tailored to their specifications at 4% of their body weight twice daily for a duration of 150 days. Despite all net cages being situated in the same earthen pond, water quality was assessed, revealing the following parameters: water temperature ranged from 28.5 to 30.2 °C, DO levels were between 6.5 and 7.3 mg/L, pH values varied from 7.15 to 8.52, and total dissolved solids were between 0.21 and 0.52 g/L.

### 2.11. Determination of Growth Performance

All the fish in each net cage were weighed at 15-day intervals to adjust feed volume, and growth parameters were then estimated with the results being put in a report at 30-day intervals. Growth indices such as weight gain (WG), average daily gain (ADG), specific growth rate (SGR), feed conversion rate (FCR), protein efficiency ratio (PER), and survival rate (SR) were determined using the following equations [25]:

WG = final weight (g) − initial weight (g) ADG = [{final weight (g) − initial weight (g)}/experimental days] SGR = [{ln final weight (g) − ln initial weight (g)}/experimental days] × 100 FCR = total feed fed (g) / weight gain (g) PER = WG (g)/crude protein fed (g) SR = [number of survival fish/initial number of fish] × 100

### 2.12. Pathogenic Challenge Test

*Aeromonas hydrophila* strain DMST 21250 was procured from the Department of Medical Sciences, Ministry of Public Health, Thailand. To obtain a fresh, actively growing culture, the strain was cultured aerobically in Tryptic Soy Broth (TSB) at 37 °C for 18 to 24 h. Physiological saline was used to dilute the bacterial suspension to a turbidity equivalent to the 0.5 McFarland standard. This turbidity matched roughly 1.0 × 10^8^ CFU/mL, as confirmed by serial dilution and colony enumeration. A new inoculum was prepared daily before injection and kept on ice to maintain bacteria viability. The virulence and pathogenicity were assessed using LD_50_ prior to the challenge test on *L. chrysophekadion*, revealing a concentration of 10^8^ CFU/mL. To investigate the bacterial resistance of *L. chrysophekadion* to *A. hydrophila*, 60 fish (20 per replication) from each group were utilized. A simple random sampling method was employed by using a handheld fish net to scoop fish from the tank until 20 individuals were obtained. Following the 150-day feeding trial, the four groups administered an intraperitoneal injection of 0.1 mL containing 10^8^ CFU/mL, whereas the negative control group received 0.1 mL of physiological saline (0.85%). The mortality assessment parameters for the bacterial challenge involved recording the death of fish in each group daily for 7 days after inoculation. The cumulative mortality rate (%) was then calculated and presented in the form of a line graph.

### 2.13. Gene Expressions

#### 2.13.1. Primers

Since the nucleotide sequences of the *IL-1β*, *IGF-1*, *CAT*, and *β-actin* genes of *L. chrysophekadion* are not available in public databases, primers for RT-qPCR amplification were used based on homologous gene sequences from the closely related species *Labeo rohita* (Table 1). To verify the identity of the target genes, two amplified sequence samples from each gene were subjected to bidirectional sequencing at ATGC Co., Ltd., Khlong Luang, Thailand. The forward and reverse sequences were aligned and assembled using the Clustal Omega program version 1.2.4 [26]. Subsequently, the consensus sequences were subjected to similar analysis using the BLASTn program [27].

#### 2.13.2. RT-qPCR Analysis

A total of six fish were randomly sampled from each treatment group (T1–T4) for gene expression analysis. Simple random sampling was conducted by using a handheld fish net to scoop two fish at a time from the tank. Following anesthesia using MS222 (Sigma, St. Louis, MO, USA) solution at a concentration of 0.2 g/L, liver tissues were collected and preserved in TRIzol reagent (Molecular Research Center, Cincinnati, OH, USA) at −20 °C until RNA extraction. One microgram of the extracted total RNA was used for first-strand cDNA synthesis using the iScript™ Select cDNA Synthesis Kit (Bio-Rad, Hercules, CA, USA), following the manufacturer’s protocol. RT-qPCR reactions were conducted in a final volume of 20 μL, comprising 10 μL of 2× master mix (THUNDERBIRD™ SYBR^®^ qPCR Mix, TOYOBO, Osaka, Japan), 0.3 μL of 10 μM of each primer, and 2 μL of first-strand cDNA (10 ng/μL). The amplification protocol included an initial denaturation at 95 °C for 1 min, followed by 40 cycles of denaturation at 95 °C for 15 s; annealing at 60 °C for 15 s for the *IL-1β*, *IGF-1*, and *β-actin* genes, and 57 °C for 15 s for the *CAT* gene; and extension at 72 °C for 10 s. A melting curve analysis was subsequently performed from 55 °C to 95 °C, with a ramp rate of 0.3 °C/s. Each sample was subjected to triplicate reactions for analysis.

The relative expression levels of the three target genes, including *IL-1β*, *IGF-1*, and *CAT*, were determined using the 2^−ΔΔCT^ method, following Ref. [31]. To account for variations in total RNA input, the threshold cycle difference (ΔCT) between each target gene and the reference gene *β-actin* was calculated for each reaction, using *β-actin* as the internal control.

### 2.14. Statistical Analysis

Statistical analyses were conducted utilizing SPSS version 25.0 (IBM Corporation, Armonk, NY, USA). The level of significance was established at *p* < 0.05. Differences among groups were analyzed using one-way ANOVA, with Tukey’s post hoc test applied for multiple comparisons. Normality of data was checked with the Shapiro–Wilk test, and the homogeneity of variances was assessed using Levene’s test. All data were expressed as mean ± standard deviation (SD).

## 3. Results

### 3.1. Phytochemical Profiles of PPE

Although several constituents were not detected, anthraquinones, phenolics, triterpenoids, and carbohydrates were successfully identified using standard phytochemical reagents (Table 2).

### 3.2. Total Phenolic Content, Total Flavonoid Content, and Antioxidant Capacity of PPE

The ethanolic extract of *P. pulcherimus* leaves contained a total phenolic content of 96.00 mg gallic acid equivalents per gram of extract. The total flavonoid content was 17.55 mg quercetin equivalents per gram of extract. The antioxidant activity, assessed via DPPH radical scavenging assay, revealed that the concentration of the test sample that produces the 50% inhibition (IC_50_) value of the PPE was 1314.08 μg/mL, which is approximately 175 times less potent than that of ascorbic acid (IC_50_ = 7.53 μg/mL). These results are presented in Table 3.

### 3.3. Growth Performance and Survival Rate

**Figure 1:**
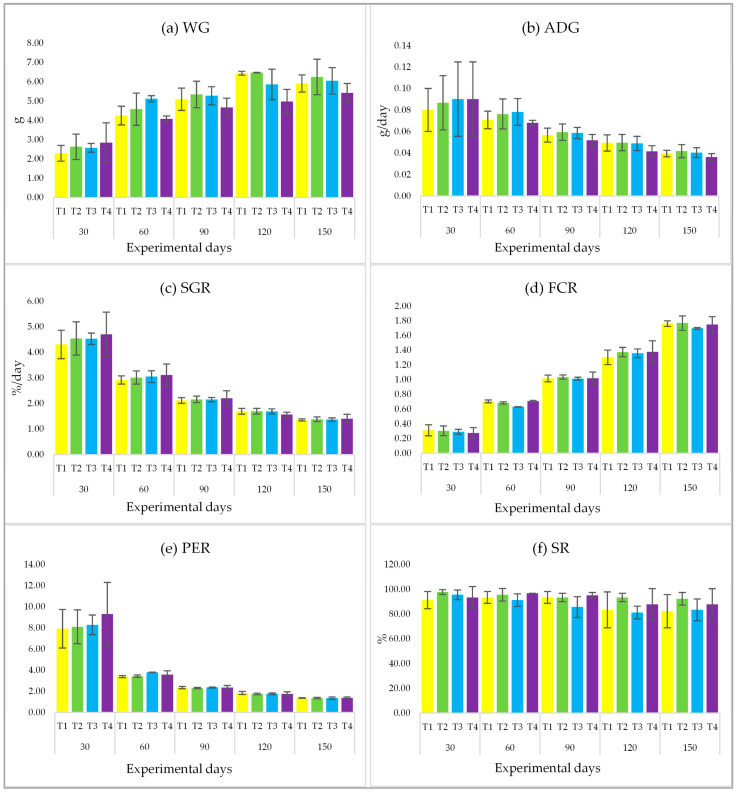
Growth indices consisting of (**a**) weight gain: WG, (**b**) average daily gain: ADG, (**c**) specific growth rate: SGR, (**d**) feed conversion rate: FCR, (**e**) protein efficiency ratio: PER, and (**f**) survival rate: SR of *Labeo chrysophekadion*, which were fed different levels of *Phlogacanthus pulcherrimus* extract. T1 (0.00 g/kg), T2 (0.25 g/kg), T3 (0.50 g/kg), and T4 (0.75 g/kg).

Throughout the 150-day experimental period of feeding with PPE at four different concentration levels (including the control group), it was found that all parameters, i.e., WG, ADG, SGR, FCR, PER, and SR, showed no statistical difference (*p* > 0.05) (Figure 1a–f).

### 3.4. Cumulative Mortality Rate Following Pathogenic Exposure

**Figure 2:**
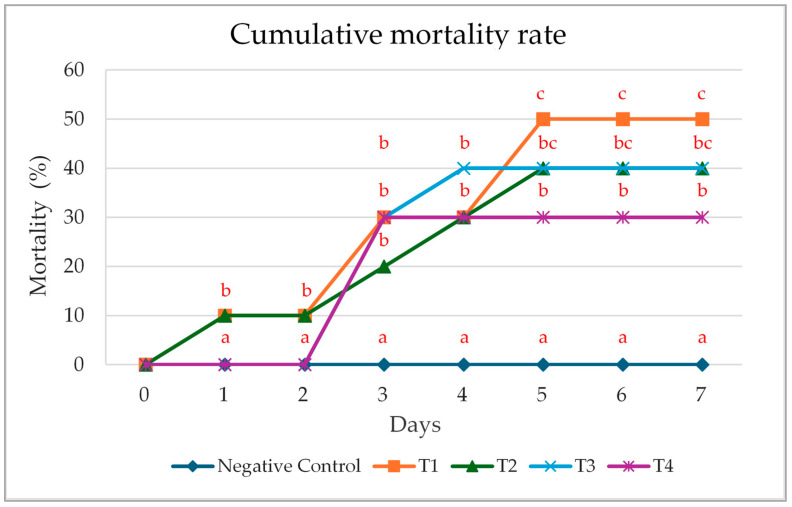
Cumulative mortality rate (%) of *Labeo chrysophekadion*, fed with the four different concentrations of *Phlogacanthus pulcherrimus* extract against *Aeromonas hydrophila* for 7 days. T1 (0.00 g/kg), T2 (0.25 g/kg), T3 (0.50 g/kg), T4 (0.75 g/kg), and negative control (0.85% NaCl). Different letters indicate statistically significant differences (*p* < 0.05) for each day.

After a 150-day experimental period, each group of test fish received an injection of *A. hydrophila* suspension at a concentration of 10^8^ CFU/mL, while the negative control group was injected solely with 0.85% saline. The injections were administered only on the first day. Subsequently, the fish were monitored for clinical symptoms, and mortality data were collected continuously over a period of 7 days. It was found that the control group experienced the highest cumulative mortality rate, with deaths starting from the first day of *A. hydrophila* exposure. Groups T2 and T3 showed identical cumulative mortality rates. The highest cumulative mortality rate in group T3 was observed on day 4, whereas that of group T2 occurred on day 5 post-injection. Group T4 had a lower cumulative mortality rate compared to groups T1, T2, and T3. As expected, the negative control group did not experience any mortality during the 7-day period after the bacterial challenge (Figure 2).

### 3.5. Relative Gene Expression Levels

Based on a comparison with sequences available in GenBank, the two amplified sequence samples from each gene were confirmed to be the target genes, showing 96–100% sequence identity with *L. rohita*. The sequences of four genes of *L. chrysophekadion* were submitted under GenBank accession number PV926119-PV926122. Therefore, the nucleotide sequences of these primers are suitable for use in gene expression studies in *L. chrysophekadion*.

**Figure 3:**
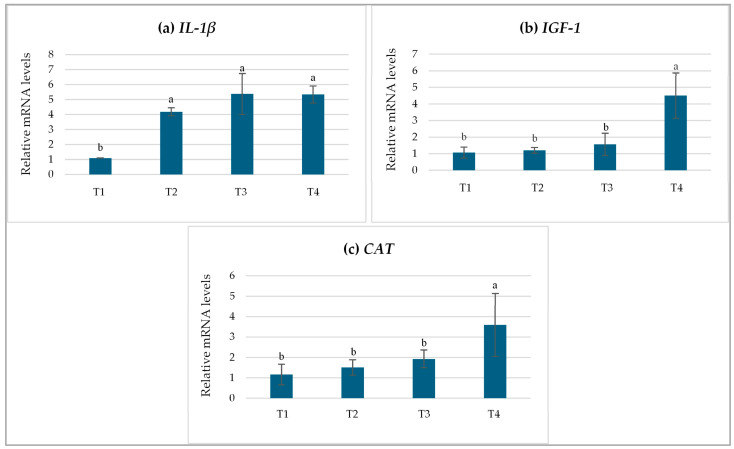
Relative expression levels of (**a**) *IL-1β*, (**b**) *IGF-1*, and (**c**) *CAT* genes in *Labeo chrysophekadion* following administration of *Phlogacanthus pulcherrimus* extract on day 150. T1 (0.00 g/kg), T2 (0.25 g/kg), T3 (0.50 g/kg), and T4 (0.75 g/kg). Results are presented as mean values ± standard deviation (SD). Different letters indicate statistically significant differences (*p* < 0.05).

On day 150, the relative expression of the *IL-1β* gene in *L. chrysophekadion* was significantly elevated (*p* < 0.05) in all groups administered PPE (T2-T4) compared to the control group (T1) (*p* < 0.05) (Figure 3a). The highest levels of gene expression were detected in groups T3 and T4, exhibiting an approximate fivefold increase relative to group T1. This was followed by group T2, which showed a fourfold increase compared to group T1. The relative expression levels of the *IGF-1* gene varied across the treatment groups. Notably, fish in group T4 exhibited the highest expression level (*p* < 0.05), with nearly a fourfold increase compared to the other groups (Figure 3b). In contrast, no significant differences were observed among the groups T1, T2, and T3. Group T4 exhibited the highest statistically significant (*p* < 0.05) relative expression of the *CAT* gene, showing a 3.5-fold increase compared to group T1 (Figure 3c). While no significant differences were detected among the groups T1, T2, and T3, the relative expression levels of both *IGF-1* and *CAT* genes in all treatment groups were elevated compared to the control.

## 4. Discussion

The phytochemical analysis of the PPE revealed the presence of several key secondary metabolites, including anthraquinones, phenolics, and triterpenoids, suggesting a diverse biochemical composition with potential biological activities [32,33]. The identification of phenolics is particularly noteworthy, as these compounds are recognized for their antioxidant and free radical scavenging properties, which could contribute to the observed protective effects against oxidative stress [34]. The total phenolic content of the ethanolic extract of *P. pulcherimus* leaves was found to be 96.00 mg GAE/g extract, indicating a significant concentration of phenolic compounds that could contribute to its antioxidant potential [35]. The total flavonoid content, another important class of phenolic compounds, was measured at 17.55 mg QE/g extract, further supporting the antioxidant capacity of the extract [36]. It is noteworthy that total flavonoid content was detected using the modified aluminum chloride colorimetric assay, albeit at a relatively low concentration. However, preliminary phytochemical screening failed to detect flavonoids. This discrepancy could be explained by two possible reasons: First, the presence of interfering substances in the extract may have affected the colorimetric reaction used in the preliminary test, leading to a false-negative result; second, the flavonoid content may have been too low to be detected by the less sensitive preliminary assay. The antioxidant capacity of the PPE, as indicated by the DPPH assay (IC_50_ = 1314.08 μg/mL), was relatively weak compared to ascorbic acid (IC_50_ = 7.53 μg/mL). This difference in potency could be attributed to the specific types and concentrations of phenolic compounds present in the extract, as well as potential synergistic or antagonistic interactions among them [37,38]. The presence of compounds like gallic and salicylic acids, as well as quercetin, could contribute to the observed antioxidant properties [39]. Polyphenols are known to modulate enzyme activity and cell receptor function, offering a range of biological actions beyond antioxidant effects in disease prevention and treatment [40,41]. Phenolic compounds have demonstrated remarkable bioactivities, including anticancer, anti-inflammatory, and antibacterial properties, as well as the ability to reduce the risk of diabetes, cardiovascular disease, and neurodegenerative diseases [42]. Furthermore, the antioxidant activity of phenolics is attributed to their ability to scavenge free radicals and modulate the production of reactive oxygen species, thereby protecting cellular components from oxidative damage [43].

While the present study did not observe significant improvements in growth parameters with *P. pulcherimus* extract supplementation, it is important to note that the effects of dietary supplements can vary depending on the specific compound, dosage, fish species, and rearing conditions. Herbal bioactive compounds, including flavonoids, alkaloids, terpenoids, and phenolics, primarily act as immunomodulators and antioxidants. Their mechanisms focus on activating immune cells such as macrophages and neutrophils, promoting cytokine production like interleukin-1β, and boosting antioxidant enzyme systems such as catalase. These compounds do not directly enhance nutrient digestibility, absorption, or anabolic metabolism [44,45]. Moreover, the dose used in this instance may be too low to observe a notable difference in growth. However, the effective dose for stimulating the immune system or antioxidant defenses is often lower than the dose required to induce significant growth response. The doses employed in many studies are typically optimized based on immune parameters or challenge tests, rather than on maximizing growth [46]. Meanwhile, higher doses intended for growth might sometimes even be immunosuppressive or cause palatability issues, counteracting potential benefits [47]. In systems that are well-managed with nutritionally balanced diets and low levels of pathogen pressure, healthy fish are likely already achieving their genetic growth potential. Under such optimal conditions, the addition of an immunostimulant may not significantly enhance growth. Instead, its main advantage emerges during challenges such as bacterial infections or stress, where the immune system’s readiness offers protection [48]. Further research may be warranted to explore the potential benefits of *P. pulcherimus* extract on growth performance under different stress conditions or in combination with other dietary additives. The improved survival rate observed in the 0.75 g/kg group suggests that *P. pulcherimus* extract may possess antibacterial or immunostimulatory properties that enhance the fish’s ability to combat *A. hydrophila* infection. Similar studies have demonstrated the protective effects of plant extracts against bacterial infections in fish, highlighting the potential of natural compounds as alternatives to antibiotics in aquaculture [49]. Furthermore, herbal–probiotic combinations have shown promise in enhancing hematological parameters, immune function, and antioxidant capacity in fish [50]. The use of herbal extracts can be a valuable component of efforts to improve fish health and reduce reliance on synthetic drugs in aquaculture [51].

The analysis of *P. pulcherrimus* extract’s influence on gene expression in *L. chrysophekadion* revealed an involved relationship of immune, growth, and antioxidant responses. The initial verification of primer specificity, demonstrating 96–100% sequence identity with target genes in GenBank, establishes an adequate foundation for subsequent gene expression analyses, promising that the observed alterations in gene expression are related to the intended targets rather than to non-specific amplification or primer mismatches [52]. The administration of *P. pulcherrimus* extract significantly affected the relative expression of immune-, growth- and antioxidant enzyme-related genes in *L. chrysophekadion*, demonstrating dose-dependent effects. The upregulation of *IL-1β* across all treatment groups compared to the control group suggests that the extract possesses immunostimulatory properties. *IL-1β* is a pro-inflammatory cytokine critical for initiating immune responses [53], and its elevated expression indicates enhanced immune activation [54]. Therefore, the extract from *P. pulcherrimus* may contribute to the enhancement of the immune response in *L. chrysophekadion*. This finding is supported by the lower mortality rates observed in fish treated with *P. pulcherrimus* extract when challenged with *A. hydrophila*, indicating enhanced disease resistance compared to the untreated control group. The *IGF-1* gene, which plays a pivotal role in growth [55], exhibited significantly higher expression only at the highest extract concentration at 0.75 g/kg. These findings indicate that *P. pulcherrimus* extract may promote growth performance at appropriate dosage levels, aligning with previous studies in other fish species where phytogenic feed additives have been shown to enhance growth through *IGF-1* upregulation. For instance, curcumin derived from *Curcuma longa* has demonstrated growth-promoting effects in tilapia (*Oreochromis mossambicus*) [56], while the combined use of limonene and thymol, as well as bay leaf (*Laurus nobilis*) aqueous extract, have similarly been reported to improve growth and *IGF-1* expression in Nile tilapia (*Oreochromis niloticus*) [57,58]. Interestingly, although *IGF-1* gene expression was elevated, various physiological and environmental factors such as reproductive maturation, acute stress, or changes in temperature can interfere with the normal relationship between *IGF-1* levels and somatic growth [59]. Similarly, *CAT* gene expression was highest at 0.75 g/kg, indicating enhanced antioxidant capacity. *CAT* is a key antioxidant enzyme involved in the detoxification of hydrogen peroxide (H_2_O_2_), a reactive oxygen species commonly generated during metabolic processes and under stress conditions. The enzyme catalyzes the conversion of H_2_O_2_ into water and molecular oxygen [60], which are non-toxic to cells. Magnesium, which is abundantly present in *P. pulcherrimus*, plays a crucial role as a cofactor for several enzymes involved in antioxidant biosynthesis [61], thereby contributing to its potential antioxidant properties. However, elevated catalase levels may also indicate that the fish are experiencing significant oxidative stress, which is not a normal physiological condition, such as exposure to heavy metals [62], pesticides [63], and thermal stress [64]. Despite this, the present study found that the trends of growth rate, survival rate, and disease resistance of fish supplemented with *P. pulcherrimus* extract were higher than those of the control group. This suggests that the tested extract concentration was non-toxic and potentially beneficial for the experimental fish.

## 5. Conclusions

This research elucidates that dietary incorporation of PPE significantly enhances disease resistance and modulates crucial immune and antioxidant gene expressions in *L. chrysophekadion*, despite having no impact on growth performance in unstressed conditions. The phytochemical composition of PPE, abundant in phenolics, flavonoids, anthraquinones, and triterpenoids, endows it with moderate antioxidant properties and pronounced immunostimulatory effects. Although growth parameters such as WG, ADG, SGR, FCR, and survival rate remained constant across varying PPE dosages, there was a dose-dependent upregulation of pivotal genes: *IL-1β* (indicative of immune activation), *IGF-1* (related to growth regulation), and *CAT* (involved in oxidative stress mitigation). The highest PPE concentration (0.75 g/kg) resulted in the most pronounced increase in *IGF-1* and *CAT* expressions and significantly decreased mortality by 20% when challenged with *A. hydrophila* and compared to the control group, highlighting PPE’s role in bolstering pathogen resistance. These findings advocate for PPE as a practical functional additive in sustainable aquaculture, particularly in enhancing immune response in economically valuable species such as *L. chrysophekadion*. However, the concentration of the extract used in this study was tested solely on *L. chrysophekadion*, a single species. When applied to other species or under different rearing conditions, the experimental outcomes may vary from those observed here. Additionally, the experiment was conducted in a real field setting, rather than in a controlled laboratory environment where environmental factors can be regulated. As a result, fluctuating weather conditions—such as rain, overcast skies, high temperatures, and cold weather—significantly influenced the fish’s feeding behavior and overall activity throughout the experimental period. Future research should investigate the efficacy of PPE under environmental stress conditions or in conjunction with probiotics to maximize its health benefits.

## Figures and Tables

**Table 1:** Primers used for reverse transcription–quantitative polymerase chain reaction analysis.

Gene	Primer Name	Sequence	Annealing Temperature (°C)	Amplicon Size (bp)	References
Immune-related genes					
Interleukin-1β (*IL-1β*)	IL-1β-qF	TTGAAGGCCGTGACACTGACT	60	114	[28]
	IL-1β-qR	GATTCCCAGGCACACAGGTT			
Growth-related genes					
Insulin-like growth factors 1 (*IGF-1*)	F	GCAAACCGACAGGCTATGGGC	60	166	[29]
	R	GTGTCTGTGTGCCGTTCCGC			
Antioxidant enzyme-related genes					
Catalase (*CAT*)	F	ACCTCTACAACGCCATCT	57	95	[30]
	R	ATTCCACTTCCAGTTCTCAG			
Housekeeping gene					
*β-actin*	F	CACTGCTGCTTCCTCCTCCTCC	60	139	[29]
	R	GATACCGCAAGACTCCATACCCAAG			

**Table 2:** Phytochemical composition of *Phlogacanthus pulcherrimus* extract.

Phytochemicals	Results	Phytochemicals	Results
Alkaloids	−	Phenolics	+
Anthocyanins	−	Flavonoids	−
Anthraquinones	+	Hydrolysable tannins	−
Steroids	−	Condensed tannins	−
Saponins	−	Carbohydrates	+
Triterpenoids	+	Cyanogenic glycosides	−
Volatile coumarins	−	Cardiac glycosides	−
Nonvolatile coumarins	−		

Note: (+) present, (−) absent.

**Table 3:** Total phenolic content, total flavonoid content, and antioxidant capacity of *Phlogacanthus pulcherrimus* extract.

Analysis	Total Phenolics (mg GAE/g Extract)	Total Flavonoids (mg QE/g Extract)	Antioxidant Capacity (IC_50_) (μg/mL)
*P. pulcherimus* extract	96.00 ± 14.58	17.55 ± 3.18	1314.08 ± 3.60
Ascorbic acid	-	-	7.53 ± 3.19

Values are presented as mean ± SD (*n* = 3). IC_50_ = the concentration of the test sample that produces 50% inhibition. The IC_50_ of *Phlogacanthus pulcherrimus* extract was extrapolated by GraphPad Prism 10.4.2 due to its limit of solubility. The SDs of antioxidant capacities were calculated using the lack-of-fit model.

## Data Availability

Data are contained within the article.

## Abbreviations

The following abbreviations are used in this manuscript:

PPE	*Phlogacanthus pulcherrimus* leaf ethanol extract
GAE	Gallic acid equivalent
QE	Quercetin equivalent
IC50	Inhibition concentration 50%
IL-1β	Interleukin-1β
IGF-1	Insulin-like growth factor 1
CAT	Catalase
CFU	Colony-forming unit
DPPH	2,2-diphenyl-1-picrylhydrazyl
RT-qPCR	Reverse transcription-quantitative polymerase chain reaction
DO	Dissolved oxygen
WG	Weight gain
ADG	Average daily gain
SGR	Specific growth rate
FCR	Feed conversion rate
PER	Protein efficiency ratio
SR	Survival rate
LD_50_	Lethal dose 50%
SD	Standard deviation
H_2_O_2_	Hydrogen peroxide
